# No Association Between Loneliness, Episodic Memory and Hippocampal Volume Change in Young and Healthy Older Adults: A Longitudinal European Multicenter Study

**DOI:** 10.3389/fnagi.2022.795764

**Published:** 2022-02-23

**Authors:** Cristina Solé-Padullés, Dídac Macià, Micael Andersson, Mikael Stiernstedt, Sara Pudas, Sandra Düzel, Enikő Zsoldos, Klaus P. Ebmeier, Julia Binnewies, Christian A. Drevon, Andreas M. Brandmaier, Athanasia M. Mowinckel, Anders M. Fjell, Kathrine Skak Madsen, William F. C. Baaré, Ulman Lindenberger, Lars Nyberg, Kristine B. Walhovd, David Bartrés-Faz

**Affiliations:** ^1^Department of Medicine, Faculty of Medicine and Health Sciences, Institute of Neurosciences, University of Barcelona, Barcelona, Spain; ^2^ISGlobal, Hospital Clínic – University of Barcelona, Barcelona, Spain; ^3^Department of Integrative Medical Biology, Umeå University, Umeå, Sweden; ^4^Umeå Center for Functional Brain Imaging, Umeå University, Umeå, Sweden; ^5^Center for Lifespan Psychology, Max Planck Institute for Human Development, Berlin, Germany; ^6^Max Planck UCL Centre for Computational Psychiatry and Ageing Research, Berlin, Germany; ^7^Department of Psychiatry, Wellcome Centre for Integrative Neuroimaging, University of Oxford, Oxford, United Kingdom; ^8^Department of Psychiatry, Amsterdam Neuroscience, Amsterdam UMC, Vrije Universiteit Amsterdam, Amsterdam, Netherlands; ^9^Vitas Ltd., Oslo, Norway; ^10^Department of Nutrition, Institute of Basic Medical Sciences, Faculty Medicine, University of Oslo, Oslo, Norway; ^11^Center for Lifespan Changes in Brain and Cognition, University of Oslo, Oslo, Norway; ^12^Department of Radiology and Nuclear Medicine, Oslo University Hospital, Oslo, Norway; ^13^Danish Research Centre for Magnetic Resonance, Centre for Functional and Diagnostic Imaging and Research, Copenhagen University Hospital Amager and Hvidovre, Copenhagen, Denmark; ^14^Radiography, Department of Technology, University College Copenhagen, Copenhagen, Denmark; ^15^Department of Radiation Sciences, Umeå University, Umeå, Sweden; ^16^August Pi i Sunyer Biomedical Research Institute (IDIBAPS), Barcelona, Spain

**Keywords:** loneliness, episodic memory, hippocampus, cortical thickness, adolescence, cognitive decline

## Abstract

**Background:**

Loneliness is most prevalent during adolescence and late life and has been associated with mental health disorders as well as with cognitive decline during aging. Associations between longitudinal measures of loneliness and verbal episodic memory and brain structure should thus be investigated.

**Methods:**

We sought to determine associations between loneliness and verbal episodic memory as well as loneliness and hippocampal volume trajectories across three longitudinal cohorts within the Lifebrain Consortium, including children, adolescents (*N* = 69, age range 10–15 at baseline examination) and older adults (*N* = 1468 over 60). We also explored putative loneliness correlates of cortical thinning across the entire cortical mantle.

**Results:**

Loneliness was associated with worsening of verbal episodic memory in one cohort of older adults. Specifically, reporting medium to high levels of loneliness over time was related to significantly increased memory loss at follow-up examinations. The significance of the loneliness-memory change association was lost when eight participants were excluded after having developed dementia in any of the subsequent follow-up assessments. No significant structural brain correlates of loneliness were found, neither hippocampal volume change nor cortical thinning.

**Conclusion:**

In the present longitudinal European multicenter study, the association between loneliness and episodic memory was mainly driven by individuals exhibiting progressive cognitive decline, which reinforces previous findings associating loneliness with cognitive impairment and dementia.

## Introduction

Loneliness is a subjective and negative emotion related to dissatisfaction with the quantity or quality of social connections (Hawkley and Cacioppo, 2010). Previous literature states that loneliness confers increased risk of all-cause mortality as well as cardiovascular disorders (Holt-Lunstad and Smith, 2016), which might be mediated by unhealthy lifestyle or depression (Holwerda et al., 2016). Indeed, people who report feeling lonely are at higher risk of depression, and, similarly, depression reinforces feelings of loneliness (Cacioppo et al., 2006). Although loneliness and social isolation are related, with the latter describing an objective state of minimal social contact or even lack of social support (Ong et al., 2016; Yanguas et al., 2018), both entities represent independent risk factors for cognitive decline and dementia with advanced age (Holwerda et al., 2014; Kuiper et al., 2015; Sundström et al., 2020; Sutin et al., 2020). While some researchers have used both terms indistinctly, more recently, they have been differentiated, although they both appear to be common predictors of social frailty and mortality (Yanguas et al., 2018).

Associations between greater loneliness and lower cognitive functioning have been found in cross-sectional studies with advanced age (reviewed in Boss et al., 2015), as well as in a previous longitudinal study with repeated measures of loneliness and cognition (Wilson et al., 2007). But loneliness does not only affect older people, and there appears to be a U-shaped relationship, where younger and older adults present the highest prevalence (Lasgaard et al., 2016). Hence, interventions to alleviate loneliness among young people have been carefully designed (reviewed in Eccles and Qualter, 2021). Studies aiming to explore associations between loneliness and cognitive functions among young populations are lacking, and loneliness and stress are entangled in a way that the former may contribute to strengthen the acknowledged implication of stress on hippocampal neurogenesis and memory formation (see review by Kim and Diamond, 2002).

Research attempting to identify structural brain correlates of loneliness have emerged with previous studies using a Voxel-Based Morphometry (VBM) approach, emphasizing that regions linked to processing of social information, empathy and emotional regulation would be particularly compromised; namely, fronto-temporal and limbic areas in both young (Kong et al., 2015; Nakagawa et al., 2015) and older adults (Cacioppo et al., 2014; Düzel et al., 2019). These results were derived from cross-sectional studies associating loneliness and brain structure without investigating how progression of loneliness may relate to brain structure in successive evaluations. Only one previous study explored prefrontal cortical thickness and loneliness associations before and after an exercise intervention in older adults aged 60–79 (Ehlers et al., 2017), failing to find any direct associations with loneliness.

Due to its involvement in learning and memory (Squire, 1991; Burgess et al., 2002) as well as emotion regulation (Santangelo et al., 2018), the hippocampus is a key brain structure to be considered, especially in older adults. A previous cross-sectional study found an association between this structure and loneliness (Düzel et al., 2019), highlighting its role in both cognitive and social processes linked to self-perception of social isolation. A very recent study also showed that individuals reporting loneliness and social isolation presented higher brain age (de Lange et al., 2021), relative to chronological age, which is acknowledged as a marker of brain integrity and health. There have been, however, no studies examining the association between repeated assessment of loneliness and the brain, and more specifically the hippocampus. In a recent review by Campagne (2019), it was argued that loneliness is related to circulating stress hormones, immune system as well as glutamate system functioning. As stated above, loneliness is very much associated with depression, anxiety and stress (Beutel et al., 2017; Campagne, 2019). Chronic stress is acknowledged to cause activation of the hypothalamic-pituitary-adrenal (HPA) axis, which leads to elevated circulating glucocorticoids (Sapolsky, 1996). The hippocampus presents a high concentration of glucocorticoid receptors and it has been demonstrated that one of the causes associated with an accelerated damage of hippocampal neurons is a prolonged high concentration of corticoids (Sapolsky, 1996). The fact that loneliness is experienced as a feeling possibly leading to mental distress further contributes to hypothesize that loneliness might also promote a stress-related chain, with overactivation of HPA axis, and possible impact on hippocampal volume trajectories, particularly among older adults. Furthermore, this putative association might also be seen among preadolescents. Wiedenmayer et al. (2006) found associations between cortisol levels and specific portions of the hippocampus morphology among children, which were positive for anterior parts and negative for lateral portions, and loneliness has been previously linked to stress (Campagne, 2019). Due to the complexity of the developing brain at these early stages, with the acknowledged initial decrease of gray matter volume and cortical thickness (Gennatas et al., 2017), it is of relevance to explore putative associations between loneliness and hippocampus among children, as compared to old adults.

In a recent review, authors stressed that large and diverse longitudinal cohorts are needed to elucidate the neurobiology of loneliness (Lam et al., 2021). In this line, it might be relevant to explore loneliness as a long-term feeling. Likewise, longitudinal investigation of brain structural and cognitive correlates of loneliness in younger and older adults, i.e., groups that exhibit the highest loneliness rates, are required to gain understanding of how loneliness may be associated with poor brain health. Furthermore, a longitudinal approach offers a unique opportunity to model how changes in loneliness status may relate to a particular cognitive and structural trajectory, and this is especially important when taking into account typically-developing children as well as older adults.

With longitudinal data from the European Lifebrain Consortium^1^ (Walhovd et al., 2018), we aimed to explore the association between loneliness and verbal episodic memory, as well as loneliness and hippocampal (HPC) volume change across young and older participants. Furthermore, we explored possible associations between loneliness and cortical thickness across the cortical mantle with no previous hypothesis, as no preceding studies had used this technique in the study of structural correlates of loneliness. Thus, we consider this analysis to be exploratory.

## Materials and Methods

### Subjects

**FIGURE 1:**
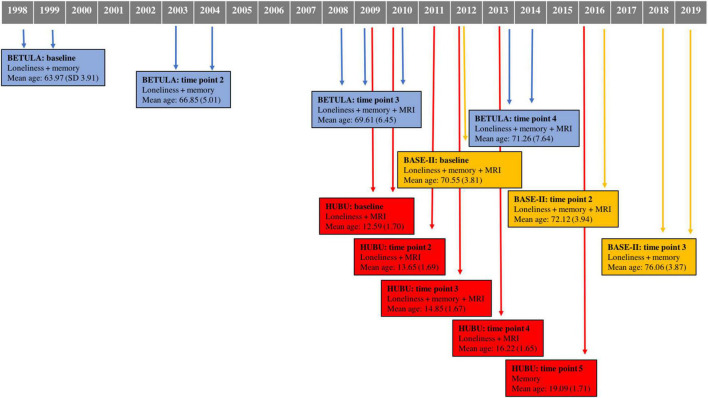
Data collection timeline for each cohort, indicating time of assessment, measures conducted for each time point, as well as mean age and standard deviation. MRI, magnetic resonance imaging.

A total of 1,537 participants were drawn from three cohorts within Lifebrain: BETULA (aged 60–85), BASE-II (aged 60–86), and HUBU (aged 10–15 at baseline). Table 1 shows further details on cohort size, waves of assessment for loneliness, verbal episodic memory and Magnetic Resonance Imaging (MRI) of the brain for each participating cohort. Figure 1 depicts timelines of assessments for each cohort, indicating the year of assessment, mean age at each particular time point and measures undertaken at each evaluation.

**TABLE 1:** Lifebrain eligible study cohorts: sample sizes and waves of assessment for each measure of loneliness, episodic memory, and magnetic resonance imaging (MRI).

Study cohort (city/country) and sample characteristics	Loneliness (*N*: sample size for each time point)	Memory (*N*: sample size for each time point)	MRI image (T1) (*N*: sample size for each time point)
BETULA (Umeå/Sweden): aged 60–85 (Nilsson et al., 1997).	Four time-points (*N* = 143, 185, 260, and 250). Interval period: 5 years.	Four time-points (*N* = 143, 185, 260, and 250). Interval period: 5 years.	Two time-points of MRI (*N* = 230 and 168). Interval period: 4 years.
BASE-II (Berlin/Germany): aged 60–86 (Bertram et al., 2014).	Three time-points (*N* = 1325, 219, and 844). Mean interval periods ranging from 2.3 to 3.23, with a mean of 5.54 years (*SD* 0.45) between first and last assessment.	Three time-points: (*N* = 1323, 218, and 749). Mean interval periods ranging from 2.3 to 3.24, with a mean of 5.57 (*SD* 0.45) between first and last assessment.	Two time-points (*N* = 215 and 215). Mean interval period: 2.29 years (*SD* 0.45)
HUBU (Copenhagen/Denmark): aged 10–15 (Madsen et al., 2018).	Four time-points (*N* = 69, 68, 59, and 39). Mean interval periods from 1.11 to 1.34, with a mean interval period of 3.43 years (*SD* 0.43) between first and last assessment.	Two time-points (*N* = 59 and 31). Mean memory interval period: 4.04 (*SD* 0.23).	Four time-points (*N* = 66, 64, 42, and 38). Mean interval periods from 1.08 to 1.35, with a mean interval period of 3.42 years (*SD* 0.45) between first and last MRI.

A subset of the BETULA cohort with data on loneliness, cognition and MRI was included in the study. Because this was a study with rolling recruitment, sample size increased at each wave of assessment (see Table 1). Exclusion criteria included severe visual or auditory impairment, intellectual or developmental disabilities, suspected dementia, having a mother tongue other than Swedish, any contraindication to MRI, neurological disorders, Mini-Mental State Examination (MMSE) < 24, brain surgery or substantial anatomical deviations (Nilsson et al., 1997; Gorbach et al., 2020). BASE-II cohort included healthy community-dwelling older adults living in the greater Berlin metropolitan area with normal or corrected to normal vision. Exclusion criteria included MMSE scores < 25, any history of psychiatric or neurological conditions or history of head injuries (Bertram et al., 2014; Gerstorf et al., 2016). Two waves of assessment with cognition and MRI were included; this latter only for a subset of 215 (see Table 1). A third follow-up was implemented by the GendAge study, with measures of loneliness and cognition, but not MRI, for the majority of the initial sample (Demuth et al., 2021).

The longitudinal HUBU cohort includes typically-developing children older than seven who were recruited from three elementary schools in the Copenhagen suburban area in 2007. Exclusion criteria included any known history of neurological or psychiatric disorders or significant brain injury, as reported by parents (Madsen et al., 2018). For these participants, it is important to note that they underwent four longitudinal assessments of loneliness and MRI, encompassing an age range from 13 to 18 in the last time-point of loneliness evaluation and thus covering a period from late childhood to late adolescence.

All volunteers had been drawn from studies where appropriate informed consent was obtained from themselves or their parents/legal guardians (Nilsson et al., 1997; Bertram et al., 2014; Madsen et al., 2018). In addition, local ethical approvals for data sharing were acquired for each participating site (Walhovd et al., 2018).

### Loneliness Measures

Loneliness scores had been obtained for each participating cohort based on the following scales. For BETULA, the following item from the Center for Epidemiological Studies – Depression scale (CES*-*D*;* Radloff, 1977) was included: “I felt lonely in the past week,” with scores ranging from 0 to 3 (‘0 – rarely or less than 1 day’ to ‘3 – most or all of the time-5–7 days’). For BASE-II, the UCLA Loneliness Scale 7 item-version was available (Russell et al., 1984). Statements such as: “I feel isolated from others,” were presented and scored on a 5-point Likert scale ranging from 1 to 5 (‘1 – strongly disagree’ to ‘5 – strongly agree’). The mean of the seven items was computed as published elsewhere (Düzel et al., 2019), with larger values indicating greater loneliness. For the HUBU cohort, an item included in the Junior Eysenck Personality Questionnaire (J-EPQ, Eysenck, 1965) was the measure taken to compute loneliness among the youngest: “Do you often feel lonely?”, which was scored from 0 to 3 (‘0 – strongly disagree’ to ‘3 – strongly agree’).

#### Harmonization of Loneliness

Since loneliness for the BASE-II cohort were derived from seven Likert-based questions with a final quantitative score, as opposed to the comparable one single Likert-based question for BETULA and HUBU, harmonization was required. The technique employed to harmonize loneliness into a qualitative variable for the three cohorts was Statistical Matching (D’Orazio et al., 2006). This method entails the assumption that distribution of loneliness scores is comparable among cohorts. In this line, it was assumed that the proportion of participants feeling high, middle and low loneliness from BETULA (also old adults) would be comparable with the ones from BASE-II. BETULA and BASE-II are both cohorts from Northern Europe and the European Commission analyzed the incidence of loneliness among European from surveys administered between 2010 and 2014. Ensuing conclusions were that ‘‘the lowest share of people who feel lonely is found in the Netherlands and Denmark (3%), Finland (4%) as well as Germany, Ireland and Sweden (5%)’’^2^. As a result, the assumption of equal distribution between BETULA and BASE-II could be accepted. The abovementioned percentage of 5% matched perfectly the one we obtained in the Swedish cohort of BETULA (5% of participants scored 2 or 3 in the question “I felt lonely in the past week”) and these two scores were joined into the category of ‘high loneliness.’ Following this, the same percentage was applied for BASE-II. Therefore, 5% of German participants with the highest scores of loneliness were classified as ‘high loneliness.’ The same was done for subjects that scored 1 (‘medium loneliness’) for BETULA, which represented 15% of the cohort. Again, the same percentage was applied for those participants from BASE-II with the highest loneliness scores, after having classified and ruled out the high loneliness group. Finally, the remaining participants were categorized as ‘low loneliness,’ which represented 80% of older participants. The loneliness scale used for the youth (HUBU) also included values ranging from 0 to 3, making it comparable to the categories described above. In this sample of older children and adolescents 13% of participants reported usually feeling lonely (score of 2 and 3: high loneliness) at baseline examination, while 40.7% felt moderately lonely (score of 1, classified as medium loneliness) and 46.3% were classified in the low group (score of 0).

The above classification of participants according to low, medium or high loneliness was applied for all time points. BETULA was again taken as a reference and since percentages of high, medium and low loneliness did not significantly change across time points, we took the same proportions (5% for high, 15% for medium, and 80% for low) for matching the remaining time points of BASE-II.

### Verbal Episodic Memory

All three cohorts included measures of verbal episodic memory, with words or sentences as items to be encoded and later retrieved. Specifically, a composite score of immediate free and cued recall of sentences was used for BETULA. More details on memory assessment are described elsewhere (Nilsson et al., 1997). For the BASE-II cohort, recognition accuracy from the Verbal Learning and Retention Test (Helmstaedter and Durwen, 1990), computed as hits minus false alarms, was used as a measure of episodic memory. For the HUBU cohort, the Verbal Affective Memory Test-26 (VAMT-26; Hjordt et al., 2020) was available, which allowed to obtain a composite score from total free and delayed recall of 10 positive, 10 negative, and 6 neutral words.

### Depressive Symptoms and Life Events

For BETULA and BASE-II cohorts, a measure of mental health status was available for each wave, matching both loneliness and memory assessments. For the former cohort, and as mentioned above, the CES-D scale was used. This scale contains 20 items scored from 0 to 3 with (maximum score of 60) and a cut-off value of 16 has been considered to identify individuals at risk of clinical depression (Lewinsohn et al., 1997). To compute depressive symptoms, we disregarded the item of loneliness, as previously reported (CES-D Minus Loneliness: CES-DML; Cacioppo et al., 2017). Baseline mean score for this cohort was 6.78 (SD: 5.74).

For the BASE-II cohort, a baseline score was available from the 15-item version of the Geriatric Depression Scale (GDS; Yesavage, 1988). Normal scores are considered from 0 to 5. The baseline mean score for the cohort was 2.17 (SD: 1.67).

For the younger sample (HUBU), a measure of exposure to negative life events was used as covariate in the statistical models. The Child and Adolescent Survey of Experiences (CASE, Allen et al., 2012) was available for all time points except for the last one, matching all loneliness measures. Thirty-four negative life events such as “family member really sick,” “been teased or bullied,” “parent split up,” or “parent lost job” amongst others were included. Maximum score was 34.

### Magnetic Resonance Imaging and Pre-processing

As detailed in Table 1, a subsample of 511 participants underwent an MRI session, including a 3D structural T1-weighted scan of the whole brain acquisition. More than one thousand observations over time were considered for the longitudinal analyses.

At each site, structural images were acquired with a 3 Tesla MRI scanner, with the following parameters: (1) BETULA: Discovery GE scanner; TR: 8.2 ms, TE: 3.2 ms, TI: 450 ms, flip angle: 12°, slice thickness: 1 mm, FoV 250 mm × 250 mm, 176 slices; (2) BASE-II: Tim Trio Siemens scanner; TR: 2,500 ms, TE: 4.77 ms, TI: 1,100 ms, flip angle: 7°, slice thickness: 1 mm, FoV 256 mm × 256 mm, 176 slices, and (3) HUBU: Magnetom Trio Siemens scanner; TR: 1,550 ms, TE: 3.04 ms, TI: 800 ms, flip angle: 9°, slice thickness: 1 mm, FOV 256 mm × 256 mm, 192 slices.

Structural brain images were processed with the longitudinal processing stream available in FreeSurfer 6.0^3^. Hippocampal (HPC) and estimated total intracranial volumes (TIV) were extracted for each time-point. All images were visually inspected for quality control.

### Statistical Analyses

To explore the associations between loneliness and age on the one hand, and loneliness and sex on the other hand, for each cohort separately, we first conducted general linear models including baseline loneliness as a categorical dependent variable, sex as a fixed factor and baseline age as a covariate of interest, with SPSS Statistics for Windows (IBM Corp. Released 2020. Version 27.0. Armonk, NY, United States: IBM Corp.). Subsequently, partial correlations between baseline loneliness and memory were performed for each cohort, adjusted for age and additionally for years of education in the older cohorts. Baseline loneliness and HPC volume associations were also examined, with age, sex, and total intracranial volume as covariates.

To examine associations between changes in verbal episodic memory or HPC volume and loneliness across time-points, statistical models were computed separately for each cohort as described below. Exploratory vertex-wise analyses associating loneliness and cortical thickness were additionally conducted (see below).

#### Loneliness and Verbal Episodic Memory/Hippocampal Volume Associations

Linear mixed-effects models run in RStudio 1.4. (RStudio Team, 2020) with the package lme4 (Bates et al., 2015), were conducted to explore associations between loneliness and verbal episodic memory and loneliness and HPC volume. We also tested a potential differential memory decline or rate of volume change for different levels of loneliness using a loneliness-by-age interaction term. The outcome variable was episodic memory or HPC volume; fixed factors included age (linear and quadratic), sex, years of education, baseline depression (for BETULA and BASE-II), categorical loneliness (as a time-varying covariate) and loneliness-by-age interaction. Change in HPC volume or change in memory was not computed prior to modeling. Interaction terms of fix effect regression with age were used to capture age-related changes. Since the age regression coefficient can be interpreted as the annual rate of change of the outcome and any interaction with it will represent an effect modifier of such expected annual rate of change of the outcome.

Within-subject random effects included intercept. We also considered adding random linear and quadratic terms to model the slope as in the fixed-effects if sufficient degrees of freedom were available (sufficient repeated measures per subject) and also a better goodness of fit was obtained (tested by comparing models with increasing number of random effects with likelihood ratio tests).

To test hypotheses involving more than one fixed effect regression coefficient, we framed them as a comparison of nested models, using a Chi squared likelihood test for inference. Fixed effect under hypothesis usually involved loneliness and its interaction with the age linear term. Significance of single fix effect regression coefficients (betas) was estimated with two-tailed t-tests using the Satterwhite approximation for the effective degrees of freedom with R package lmerTest (Kuznetsova et al., 2017).

The HUBU cohort included two time-points with verbal episodic memory performance data only overlapped with one of the four time-points in which loneliness was assessed; hence a multiple regression model was implemented with the difference of verbal episodic memory performance as a dependent variable and baseline loneliness, age, sex and negative life events as independent variables. The model with hippocampal volume as outcome, for this younger cohort included as fixed effects: age (linear and quadratic), negative life events, sex and categorical loneliness (all variables measured at each time point).

#### Loneliness and Cortical Thickness Associations

The same approach using linear mixed models as described above was used to examine associations between loneliness and cortical thickness at each vertex of extracted cortical surfaces. To account for spatial correlation of the vertex-wise tests, we used Freesurfer’s LME toolbox (Reuter et al., 2012; Bernal-Rusiel et al., 2013), for each cohort separately, with an FDR-corrected significance threshold of *p* < 0.05 for multiple comparisons.

## Results

### Baseline Associations Between Loneliness and Age, and Loneliness and Sex

Table 2 lists main demographic variables from the three included Lifebrain cohorts, as well as associations between loneliness and age and loneliness and sex at baseline examination. As shown in Table 2, there was a positive association between age and loneliness in the BASE-II cohort, with older volunteers feeling lonelier. No associations were observed for the BETULA or HUBU cohorts.

**TABLE 2:** Baseline demographics for each participating cohort and associations with loneliness derived from an ANOVA with loneliness as the outcome variable and including sex and age in the same model.

Lifebrain Cohorts	Baseline age: mean (*SD*)	Sex (% female/ % male)	Years of education: mean (*SD*)	Baseline MMSE: mean (*SD*)	Loneliness ∼ age *F*-/*p-*value	Loneliness ∼ sex *F*-/*p-*value	Loneliness ∼ memory *r*-/*p-*value	Loneliness ∼ HPC volume *r*-/*p-*value
BETULA	63.97 (3.91)	57.34/42.66	10.06 (3.90)	28.07 (1.43)	2.47/0.12	1.09/0.29	−0.12/0.19	−0.08/0.21
BASE-II	70.55 (3.81)	50.6/49.4	14.14 (2.88)	28.64 (1.18)	5.99/0.01*	4.93/0.026** (male > female)	−0.03/0.28	−0.01/0.83
HUBU	12.59 (1.70)	62.32/37.68	–	–	0.97/0.33	5.36/0.024*** (female > male)	−0.16/0.23	−0.05/0.7

*Beta = 0.011, std. error = 0.005, partial eta squared = 0.005; **beta = −0.76, std. error = 0.034, partial eta squared = 0.004; ***beta = −0.49, std. error = 0.21, partial eta squared = 0.08. Further partial correlations with baseline episodic memory and hippocampal volume (HPC) are also shown. MMSE, Mini-Mental State Examination.

For BETULA, a *post hoc* analysis considering all observations was carried out to capture the component of trajectories for both age and loneliness variables. By doing this, the association improved, with a trend toward increased loneliness with increasing age (χ^2^ = 4.9, *p* = 0.08).

Sex differences were seen within the BASE-II cohort, with males reporting significantly more feelings of loneliness than females (males: *N* = 671, mean = 1.60, *SD* = 0.62; females: *N* = 654, mean = 1.52, *SD* = 0.64). We also observed significant sex differences in loneliness in the younger cohort (HUBU), with female participants having higher scores of loneliness at baseline than males (females: *N* = 41, mean = 0.85, *SD* = 0.93; males: *N* = 24, mean = 0.33, *SD* = 0.56). No sex differences in baseline loneliness were observed for BETULA.

### Baseline Associations of Loneliness With Verbal Episodic Memory, Hippocampal Volume and Cortical Thickness

While at baseline, all correlations between loneliness and episodic memory, as well as loneliness and HPC volume were negative for all participating cohorts, they were not statistically significant (see Table 2). Likewise, in the baseline, i.e., cross-sectional, analysis of associations between loneliness and cortical thickness across the cortical mantle, none of the vertices survived FDR correction. Uncorrected results for baseline loneliness and thickness associations are depicted in the Supplementary Figure 1.

### Longitudinal Loneliness and Verbal Episodic Memory Change

**FIGURE 2:**
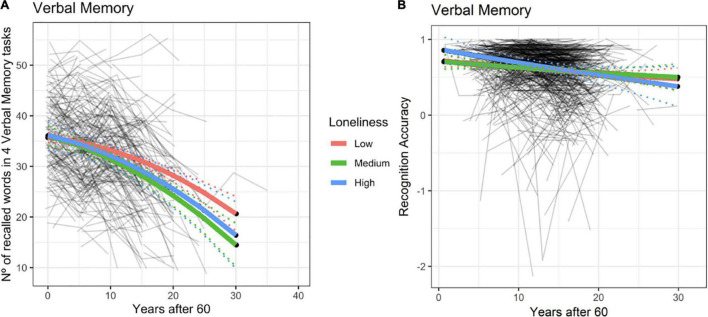
Episodic memory predicted trajectories versus age after 60 for different loneliness levels for Betula **(A)** and BASE-II **(B)**. Association between loneliness and episodic memory change within the BETULA cohort: no effect was seen at age 60 (intercept), but from then onwards, medium and high loneliness scores were related to poorer memory performance over time. Dotted lines show 95% confidence interval for predictions with random effects set to 0.

We observed a significant effect of loneliness on verbal episodic memory for BETULA (χ^2^ = 11.25, df = 4, *p* = 0.02). Such effect was clearly driven by the loneliness-by-age interaction effect with a higher degree of loneliness being associated with poorer memory performance, only at advanced ages (Figure 2A and Supplementary Table 1). This result entailed that with advancing age, having medium to high levels of loneliness was associated with decreased memory slope. Therefore, if one subject felt lonely at baseline and these feelings of loneliness were stable across time, their memory would be expected to decrease more than one subject never feeling lonely at all. Likewise, the model also predicted that if loneliness levels decreased at some point (being high at baseline and low at posterior evaluations), then memory levels would ‘normalize’ or the subject would ‘jump’ from the steeper decreased memory slope (Figure 2A, blue or green slopes) to a ‘normal’ slope of memory decrease, allegedly expected by age (Figure 2A, red slope). Therefore, the association between loneliness and memory progress would mainly be seen for those subjects with persistent feelings of loneliness. It is, however, important to note that only medium loneliness reached significant results (see Supplementary Table 1), even though the decline in episodic memory can also be seen among subjects categorized with high loneliness. It is likely that these observations may reflect fewer numbers of participants classified as high loneliness as the point estimate of the loneliness-by-age interaction term was of the same order and in the same direction for both loneliness levels.

No evidence for such effects were seen in BASE-II (χ^2^ = 1.3, df = 4, *p* = 0.86, see Figure 2B and Supplementary Table 2). Likewise, using the original measure of loneliness for this cohort and its interaction term with age did not improve the model significantly (χ^2^ = 1.20, df = 2, *p* = 0.57).

Analyses were repeated for both BETULA and BASE-II after having ruled out eight and one subject, respectively, who had developed dementia in any of the subsequent waves of assessments. For BETULA, the previous association found between memory and loneliness-by-age interaction was not significant anymore: χ^2^ = 6.62, df = 4, *p* = 0.16. For BASE-II results remained minimally changed: χ^2^ = 1.18, df = 4, *p* = 0.56.

Regression model for HUBU did not yield any significant result (*F* = 0.87, df = 4, *p* = 0.50). Supplementary Tables 1–3 provide estimates for the fixed effects of the association between verbal episodic memory and loneliness for all cohorts.

### Longitudinal Loneliness and Hippocampal Volume Change

**FIGURE 3:**
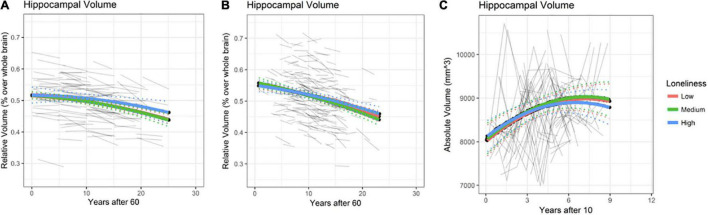
Hippocampal (HPC) volume predicted trajectories versus age after 60 for different loneliness levels for BETULA **(A)** and BASE-II **(B)**. **(C)** Depicts hippocampal trajectories for the younger participants (HUBU cohort) from age 10 onwards, as regards the loneliness variable. Raw hippocampal volumes are shown for HUBU, as absolute values might be more convenient in longitudinal studies of brain development (Goddings et al., 2014). Dotted lines show 95% confidence interval for predictions with random effects set to 0.

Rates of annual HPC volume loss from age 60 onwards are depicted in Figure 3 (at age 60 estimated 0.2% for BETULA and 0.4% for BASE-II; at age 80 these were increased to 0.47 and 0.75% for each cohort, respectively). No association between loneliness and HPC volume, as well as loneliness and HPC volume change (loneliness-by-age interaction), where intercept value (0) corresponds to age 60, was found for BETULA (χ^2^ = 4.27, df = 4, *p* = 0.36; Figure 3A) and BASE-II (χ^2^ = 2.31, df = 4, *p* = 0.68; Figure 3B). Likewise, we did not find any associations between loneliness and HPC volume changes in the younger cohort (HUBU: χ^2^ = 2.33, df = 4, *p* = 0.68). As seen in Figure 3C, for this latter cohort, HPC volume increased from age 10 to 14/15 and was followed by stabilization, emulating the non-linear developmental pattern of increased volume during late childhood and early adolescence accompanied by a slight subsequent deceleration, as described elsewhere (Tamnes et al., 2018). Supplementary Tables 4–6 provide estimates for the fixed effects of the association between hippocampal volume and loneliness, as well as loneliness-by-age interaction for BETULA, BASE-II and HUBU cohorts.

Again, re-analysis disregarding the eight participants who developed dementia along the study (8 for BETULA) yielded no significant changes on the abovementioned statistical outputs: BETULA: χ^2^ = 4.30, df = 4, *p* = 0.37. For BASE-II, the participant with onset of dementia at time point 2, was missing at that particular time point, and did not have MRI data on time point 3, so analyses were not repeated.

### Longitudinal Loneliness and Cortical Thickness Change

Longitudinal analyses of associations between loneliness and cortical thickness across the cortical mantle did not yield any statistically significant vertex-wise results after correction for multiple comparisons (FDR < 5%), in any of the cohorts. Uncorrected results are further described in the Supplementary Figure 1.

## Discussion

To our knowledge, this is the first multicenter European study to incorporate measures of loneliness over time to explore its cognitive and structural brain correlates in distinct samples across the lifespan, covering periods of late childhood, adolescence, and older adulthood.

Significant associations between age and loneliness were observed within the German (BASE-II) cohort, with older participants reporting increased feelings of loneliness; a result that is in line with the acknowledged increase of loneliness in late life, a period where one may experience a number of losses, from the death of a spouse or friends to social disengagement (Singh and Misra, 2009). Despite no baseline age and loneliness associations were seen for the BETULA cohort, a trend toward more loneliness with increasing age was seen after taking into account all observations.

We also found that German male participants presented more feelings of loneliness than their female counterparts. Notably, these participants were born between 1927 and 1953, a generation of working men and ‘stay-at-home’ women. Therefore, men were more likely to experience the life-changing event of retiring from work, and this may have intensified their feelings of loneliness, as compared to women. Yet, the above associations were not seen for the Swedish cohort of older adults (BETULA). This might be partially explained by the fact that the German cohort included more than one thousand participants at baseline examination, compared to the 143 volunteers for the BETULA cohort. This larger statistical power may have favored the emergence of significant loneliness-age and loneliness-sex associations within BASE-II. It was somehow unexpected to find more feelings of loneliness among German men, as compared to their female counterparts, because older women usually report increased loneliness (Beal, 2006; Jarach et al., 2021). Despite this, it has been recently argued that loneliness is more associated with health, functional limitations and depression (Jarach et al., 2021), than with social isolation itself, and we have not controlled for physical health variables in the present study, neither have we taken into account other generational factors (alleged ‘working men’ versus ‘stay-at-home women’), which may have contributed to the abovementioned associations found in BASE-II.

On the other hand, in the younger cohort, females reported feeling lonelier than males, a finding that is in line with a recent study, also with Danish adolescents (Eccles et al., 2020). Authors pointed to the possibility of a sex-based stigma when reporting feelings of loneliness among male adolescents. No sex differences were seen for the BETULA cohort, but bearing in mind the few proportion of people feeling lonely and the smaller sample size as compared to BASE-II, it is plausible that sex differences, if any, would have emerged with a more representative sample of lonely people.

Regarding associations between loneliness and episodic memory over time, we found that for BETULA, those participants reporting medium to high levels of loneliness over time displayed more memory loss at follow-up examinations, even though memory performance was not associated with loneliness at baseline. Thus, self-perceived and constant feelings of loneliness among these participants co-occurred with a decrease in memory performance for this cohort of Swedish participants. It is worth mentioning, however, that the model was conducted in a way that loneliness at one time point predicted memory for that particular time point, without taking into account previous loneliness levels. Despite this, interpretations considering distinct trajectories of loneliness are possible and this model predicted that low baseline loneliness for BETULA participants, combined with increases of loneliness feelings in any subsequent evaluation would be equally associated with steeper episodic memory decline, for that particular time point. Therefore, persistent loneliness but also increases in loneliness perceptions, were associated with episodic memory loss among this cohort of participants. In a previous review discussing associations between loneliness and global cognitive function as well as episodic memory, Boss et al. (2015) suggested that some associations may disappear after adjusting for demographic and psychosocial factors that influence loneliness. In our study, the association between loneliness and memory performance over time among BETULA participants was seen while controlling for age, education, sex and depressive symptoms, which emphasizes the relevance of loneliness for memory maintenance in aging and reinforces the notion that loneliness can be considered a different entity from depression, as acknowledged also in previous studies examining loneliness associations with cognitive decline (Wilson et al., 2007). Nonetheless, the association was lost after having ruled out the eight cases of dementia with onset in any of the subsequent follow-up assessments. This fact reinforced previous evidence suggesting that there is an association between loneliness and cognitive impairment (Wilson et al., 2007; Sundström et al., 2020; Sutin et al., 2020). Notably, the fact that the association did not remain significant does not imply that there is no association between loneliness and memory decline, but rather that if cases of significant memory decline are excluded, then this association is critically diminished. It is important to point out the possibility of reverse causality. Loneliness may cause memory decline but memory decline might also contribute to increased feelings of loneliness. Previous data supported the notion of a bidirectional effect between cognitive ability and loneliness among older adults (Zhong et al., 2017; Okely and Deary, 2019).

As regards the lack of association between loneliness measures and memory change in the BASE-II cohort, even when using the original continuous measure of loneliness, we believe that two main factors could be contemplated. First, the verbal memory test (recognition accuracy) was not a comparable measure with the recall (free and cued) that was used for BETULA. While recall has been consistently associated with hippocampus (Aggleton and Shaw, 1996), the role of this structure on recognition has been more debated. Recognition can depend on recollection, familiarity or both processes (Yonelinas, 2002), and though some believe that the hippocampus exclusively subserves recollection, more recent data is available indicating that both recollection and familiarity are majorly supported by the hippocampus (Merkow et al., 2015). Yet, memory measures used for the two cohorts of older adults may not be completely comparable. Second, it is possible that the considerable number of drop-outs, particularly at the second wave of assessment, may have constrained our analyses. After a thorough analysis on missing data for BASE-II cohort, it was seen that a greater proportion of missing participants at time point 3, presented increased baseline loneliness (26.82% of missing volunteers at time point 3 had medium to high scores of loneliness at baseline versus 20.74% of not missing participants; χ^2^: 6.58, *p* = 0.037). Likewise, they were also older at baseline examination [mean age: 71.24 (*SD*: 4.01) versus 70.16 (3.64), *t* = 5.02, *p* < 0.0001]. On the whole, if older and more lonely participants dropped out from the study, this could represent a ‘missing not at random’ (MNAR) case, where it is likely that these participants were experiencing some kind of cognitive impairment. Assuming that the drop-outs could contain a great proportion of cognitively impaired patients and bearing in mind that the association between loneliness and memory decline was stronger in BETULA when the eight cases of dementia were included, it is reasonable to think that the MNAR could be masking some associations.

We did not find any associations between loneliness and difference in memory performance for the HUBU cohort. While loneliness was measured at four time points, memory testing was only administered in two waves of assessments, and only one of them co-occurred with the loneliness measure. An increased number of cognitive measurements would have been ideal to better grasp memory trajectories and possibly capture putative associations with loneliness trajectories.

Contrary to expectations, we did not observe any statistically significant associations between loneliness and HPC volume in any of the cohorts, neither at baseline nor longitudinally. The same applied for our explorative investigations for associations between loneliness and vertex-wise cortical thickness. Our null findings are in apparent contrast with previous reports of cross-sectional loneliness-brain structure associations in healthy young and older adults (Kanai et al., 2012; Kong et al., 2015; Düzel et al., 2019). Studies with young adults pointed to compromised left frontotemporal regions (Kanai et al., 2012; Kong et al., 2015), whereas Düzel et al. (2019) found decreased volume of subcortical regions and cerebellum among lonely old adults. Although more studies are required to further investigate a putative cortical versus subcortical association of loneliness across aging groups, it is important to note that risk factors for loneliness are not comparable across life stages. For instance, a recent study with Finnish adolescents and young adults reveled that this negative feeling is related to social transitions and expectations, group differences, former destructive experiences or negative self-image among others (Sundqvist and Hemberg, 2021); while for middle-aged and older adults the most important factors contributing to loneliness are the loss of a spouse, frequency of contact with significant friends or family and the number of voluntary groups one is engaged to Cacioppo et al. (2015). It is also important to note that while the abovementioned studies focusing on cross-sectional associations between loneliness and brain structure used Voxel-Based Morphometry (VBM), we had a dual and different focus with a longitudinal approach: first, the hypothesis driven approach targeting the hippocampus as a subcortical structure related to emotional processing and episodic memory; and second, the exploratory approach of vertex-wise cortical thickness. Thus, the association between loneliness and memory decline in one cohort and lack of structural brain correlates suggests that structural correlates of loneliness may either appear later than cognitive correlates, or that the techniques employed were not sensitive enough to capture structural changes that were possibly too small to be detected with the current sample size.

Cacioppo et al. (2017) suggested that focus of attention is changed among people with long-term feelings of loneliness, and, though speculative, we believe that the result obtained for BETULA regarding the association between loneliness and poorer memory over time, might be partially explained by decreased attentional resources that might in turn affect encoding and retrieval of memories, overall resulting in poorer performance over time, even in the absence of brain structural changes. However, we cannot rule out the possibility that factors interacting with loneliness such as personality traits, social network and empathy in both young (Kanai et al., 2012; Kong et al., 2015) and older adults (von Soest et al., 2020), which have not been considered here, might account for lack of results in the other cohorts or the absence of structural correlates of loneliness among our participants.

The link between loneliness and cognitive decline has been previously established (Holwerda et al., 2014), although the nature of this association is poorly understood (Boss et al., 2015). Likewise, loneliness has a notable association with global health with a complexity that is not yet fully grasped (Yanguas et al., 2018). As already stated, a variety of mediating intrinsic and extrinsic factors interact with one another to converge in a combination of social vulnerability and frailty that might lead to a particular feeling of loneliness. More recently, it has been pointed out that the complexity of associations between loneliness and adverse health outcomes depend on a combination of interlinked genetic, social behavioral, physical and socioeconomic factors (de Lange et al., 2021).

Another issue that should be noted is the fact that only a low proportion of older participants from our study reported high levels of loneliness. While this is comparable with previous studies (Victor and Yang, 2012), it may have constrained our analyses. Differences in loneliness across European countries have been described and the European Commission’s Joint Research Centre concluded from surveys conducted in 2010, 2012, and 2014 that Eastern and Southern Europeans feel lonelier and are more socially isolated than Western and Northern Europeans (D’Hombres et al., 2018). Notably, Northern Europeans appear to tolerate greater rates of social isolation without having an impact on subjective measures of loneliness. Greater satisfaction with the social network has also been reported in the Scandinavian countries (Sundström et al., 2009). The three cohorts included in the present study come from Sweden, Germany, and Denmark, and this may account for the fact that only a very low percentage of our participants were categorized as experiencing ‘high loneliness.’ Another explanation would be the fact that depression was an exclusion criterion in the present study, and even though depression is considered a different entity from loneliness, they are mutually reinforcing (Cacioppo et al., 2006).

Taken together, abovementioned data may suggest different associations of loneliness and brain health across European regions and this study only included cohorts from Northern Europe. To understand differences between European regions it is important to note that the association between well-being and social network is complex and may include not only quantity and quality of relationships with family and friends, but also perceived social support (Berkman et al., 2000), as well as access to health resources, which may vary across European countries.

Several limitations should be considered in addition to the low number of participants with high feelings of loneliness already mentioned. Differences among cohorts, particularly, old adults should be also considered. As shown in Figure 1, there is a difference in age to consider, with BETULA participants being slightly younger than BASE-II’s at baseline examination. It is possible, in accordance with previous reports associating loneliness with increased risk of dementia (Sutin et al., 2020), that these associations are more evident in earlier stages of cognitive decline, which would favor BETULA’s association and not BASE-II’s. There was also a considerable time lag for loneliness evaluation for BETULA and BASE-II. In this line, BETULA started assessments in 1998, a period with no influence of digital technology, and were extended until 2014. As for BASE-II, first assessment took place in 2011. The way that one interacts with other people has considerably changed these past 15 years even though this may have mainly affected young people, for whom the use of social media has become an important part of their daily lives (Ryan et al., 2017). However, we cannot rule out the possibility that the feelings of loneliness measured during the last decade have been somehow influenced by digital revolution also among old adults. Further, heterogeneity of measures of episodic memory and loneliness should also be taken into account, as mentioned above. While BETULA’s memory measures included a composite of verbal free and cued recall, BASE-II’s was based on a recognition memory test. Though normality was not met for this subtest at baseline, this measure presented the best goodness of fit with age as a linear effect with the expected negative slope of memory decrease with time as compared to other memory measures such as delayed recall. It is, however, possible that this measure of memory was not sensitive enough to detect a clear memory change, as expected for age. Additionally, it would have been ideal to focus on other cognitive domains other than memory, particularly executive functions, with specific focus on attention, which may be influenced by stress-related processes (Girotti et al., 2018). As regards loneliness, measures were based on a personality or depression scale item for two cohorts, whereas the remaining cohort included a more quantitative measure, with a short form of the UCLA-loneliness scale. However, these are all accepted measures of loneliness (Yanguas et al., 2018) and were accordingly harmonized. Also, interval periods between evaluations, particularly for MRI, might be too short for BASE-II and HUBU cohorts, and this may have also been a disadvantage when exploring structural brain changes. Finally, two main points should be considered for the youngest cohort. First, while loneliness and MRI were conducted periodically at four time points, only two assessments of memory were available. Second, sample size for HUBU was not comparable to the older adults’, a fact that may have limited representativeness.

## Conclusion

We found associations between longitudinal measures of loneliness and verbal episodic memory change within one of two cohorts of healthy older adults, which was dependent on participants’ cognitive status. After excluding dementia cases, the previous association was no longer significant, strengthening previous findings associating loneliness with cognitive impairment and dementia. This study suggests that the association between loneliness and memory decline might be independent of hippocampal volume change or changes in cortical thickness. Likewise, while incidence of loneliness was increased for the younger cohort, no correlates of memory or brain structure were evidenced. We believe this is a first step toward other longitudinal approaches examining both cognitive and structural brain correlates of loneliness among healthy individuals at different life stages. Forthcoming studies would benefit from including other European countries in an ideal design able to identify ‘stable’ versus ‘changing’ feelings of loneliness and with the possibility to examine further cultural differences of self-perceived social isolation, resilience, and association of loneliness with brain health.

## Data Availability Statement

The data analyzed in this study is subject to the following licenses/restrictions: data may be made available upon reasonable request, given appropriate ethical and data protection approvals. Requests to access these datasets should be directed to https://www.lifebrain.uio.no/about/contact.

## Ethics Statement

Local ethical approvals for data sharing were acquired for each participating site (Walhovd et al., 2018). Written informed consent to participate in this study was provided by the participants’ legal guardian/next of kin.

## Author Contributions

CS-P contributed to conceptualization, data curation, formal analysis, and writing (original draft). DM and MA contributed to software, formal analysis, and visualization. MS, SP, and SD contributed to resources and data curation. EZ, KE, and WB contributed to conceptualization and writing (review and editing). JB and CD contributed to writing (review and editing). AB and AF contributed to validation and writing (review and editing). AM contributed to resources and software. KM contributed to data curation and writing (review and editing). UL contributed to resources. LN contributed to validation, resources, and writing (review and editing). KW and DB-F contributed to conceptualization, supervision, writing (review and editing), and project administration. All authors contributed to the article and approved the submitted version.

## Conflict of Interest

CD is a founder, stockowner, board member, and consultant in Vitas Ltd. The remaining authors declare that the research was conducted in the absence of any commercial or financial relationships that could be construed as a potential conflict of interest.

## Publisher’s Note

All claims expressed in this article are solely those of the authors and do not necessarily represent those of their affiliated organizations, or those of the publisher, the editors and the reviewers. Any product that may be evaluated in this article, or claim that may be made by its manufacturer, is not guaranteed or endorsed by the publisher.

## Footnotes

1
https://www.lifebrain.uio.no/

2
https://ec.europa.eu/jrc/en/news/how-lonely-are-europeans

3
https://surfer.nmr.mgh.harvard.edu/
