# Supplementary material for: No Association Between Loneliness, Episodic Memory and Hippocampal Volume Change in Young and Healthy Older Adults: A Longitudinal European Multicenter Study
Source: Front Aging Neurosci. 2022 Feb 23;14:795764. doi: 10.3389/fnagi.2022.795764 (PMC8905540; doi:10.3389/fnagi.2022.795764)
Supplement: Supplementary file 1 [file Image_1.pdf]

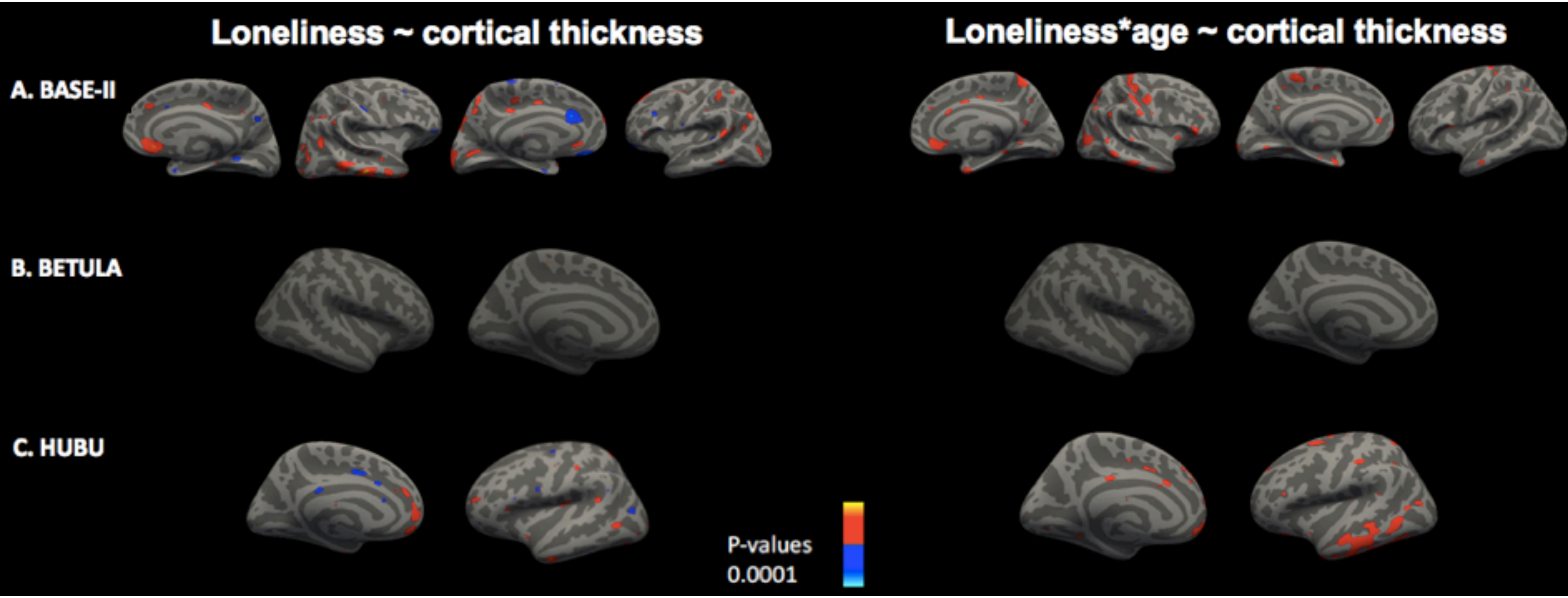

Loneliness ~ cortical thickness
Loneliness*age ~ cortical thickness
A. BASE-II
B. BETULA
C. HUBU
P-values
0.0001
