# Supplementary material for: No Association Between Loneliness, Episodic Memory and Hippocampal Volume Change in Young and Healthy Older Adults: A Longitudinal European Multicenter Study
Source: Front Aging Neurosci. 2022 Feb 23;14:795764. doi: 10.3389/fnagi.2022.795764 (PMC8905540; doi:10.3389/fnagi.2022.795764)
Supplement: Supplementary file 2 [file Data_Sheet_1.docx]

**Supplementary Figure 1.** Vertex-wise associations between loneliness and cortical thickness (left) and loneliness-by-age interaction with cortical thickness (right) for each cohort separately. Uncorrected p-values with p>0.05 (i.e. -log(0.05)<1.3) masked out. Red indicates more thickness associated with more loneliness. Blue depicts less thickness associated with more loneliness. None of the depicted clusters survived adjustment for multiple comparisons (FDR<0.05).
