# Supplementary material for: No Association Between Loneliness, Episodic Memory and Hippocampal Volume Change in Young and Healthy Older Adults: A Longitudinal European Multicenter Study
Source: Front Aging Neurosci. 2022 Feb 23;14:795764. doi: 10.3389/fnagi.2022.795764 (PMC8905540; doi:10.3389/fnagi.2022.795764)
Supplement: Supplementary file 3 [file Table_1.DOCX]

|  | Estimate | Std. Error | df | t value | Pr(>\|t\|) |
| --- | --- | --- | --- | --- | --- |
| MAIN EFFECTS | | | | | |
| (Intercept) | 39.86 | 1.19 | 353.24 | 33.53 | 0.00 |
| Education | 0.62 | 0.08 | 311.88 | 7.25 | 0.00 |
| Sex | -2.94 | 0.72 | 305.88 | -4.09 | 0.00 |
| Depression | 0.12 | 0.24 | 686.77 | 0.51 | 0.61 |
| Age_from60 | -0.08 | 0.09 | 494.69 | -0.90 | 0.37 |
| Age_from60^2^ | -0.02 | 0.00 | 424.37 | -3.78 | 0.00 |
| Loneliness_medium | 0.18 | 0.97 | 576.57 | 0.19 | 0.85 |
| Loneliness_high | 0.56 | 1.52 | 548.32 | 0.37 | 0.71 |
| INTERACTION WITH AGE | | | | | |
| Loneliness_medium | -0.20 | 0.09 | 562.60 | -2.29 | 0.02 |
| Loneliness_high | -0.17 | 0.14 | 421.35 | -1.21 | 0.23 |

**Supplementary Table 1.** Estimates for the fixed effects, with verbal episodic memory as outcome variable for the BETULA cohort. df=degrees of freedom.

|  | Estimate | Std. Error | df | t value | Pr(>\|t\|) |
| --- | --- | --- | --- | --- | --- |
| MAIN EFFECTS | | | | | |
| (Intercept) | 0.78 | 0.03 | 1830.65 | 28.75 | 0.00 |
| Education | 0.06 | 0.01 | 1078.44 | 6.92 | 0.00 |
| Sex | -0.09 | 0.02 | 1078.54 | -5.55 | 0.00 |
| Depression | -0.01 | 0.01 | 1210.75 | -1.06 | 0.29 |
| Age_from60 | -0.01 | 0.00 | 1946.75 | -5.06 | 0.00 |
| Age_from60^2^ | -0.00 | 0.00 | 1989.12 | -1.92 | 0.06 |
| Loneliness_medium | -0.05 | 0.06 | 1899.16 | -0.82 | 0.41 |
| Loneliness_high | 0.09 | 0.12 | 1930.92 | 0.73 | 0.47 |
| INTERACTION WITH AGE | | | | | |
| Loneliness_medium | 0.00 | 0.00 | 1885.22 | 0.72 | 0.47 |
| Loneliness_high | -0.01 | 0.01 | 1910.29 | -0.82 | 0.41 |

**Supplementary Table 2.** Estimates of fixed effects, with verbal episodic memory as outcome variable for the BASE-II cohort.

|  | Unstandardized beta | Std. Error | Standardized beta | t value | Pr(>\|t\|) |
| --- | --- | --- | --- | --- | --- |
| MAIN EFFECTS | | | | | |
| (Intercept) | 6.71 | 19.48 | - | 0.34 | 0.73 |
| Sex | 2.99 | 4.79 | 0.12 | 0.61 | 0.55 |
| Age | -0.17 | 1.28 | -0.03 | -0.14 | 0.90 |
| Loneliness | 1.87 | 3.86 | 0.09 | 0.48 | 0.63 |
| Negative life events | -1.41 | 0.84 | -0.32 | -1.68 | 0.11 |
| INTERACTION WITH AGE | | | | | |

**Supplementary Table 3.** Coefficients for the regression model, with difference in memory performance as outcome variable for the HUBU cohort.

|  | Estimate | Std. Error | df | t value | Pr(>\|t\|) |
| --- | --- | --- | --- | --- | --- |
| MAIN EFFECTS | | | | | |
| (Intercept) | 0.56 | 0.01 | 306.82 | 50.32 | 0.00 |
| Education | 0.0003 | 0.0008 | 277.49 | 0.38 | 0.71 |
| Sex | -0.0305 | 0.0067 | 260.88 | -4.52 | 0.00 |
| Depression | -0.0003 | 0.0013 | 153.37 | -0.22 | 0.82 |
| Age_from60 | -0.0010 | 0.0006 | 149.45 | -1.83 | 0.07 |
| Age_from60^2^ | -0.0001 | 0.0000 | 140.64 | -3.10 | 0.00 |
| Loneliness_medium | 0.0012 | 0.0052 | 148.77 | 0.23 | 0.82 |
| Loneliness_high | 0.0017 | 0.0121 | 157.45 | 0.14 | 0.89 |
| INTERACTION WITH AGE | | | | | |
| Loneliness_medium | -0.0002 | 0.0005 | 156.08 | -0.33 | 0.74 |
| Loneliness_high | 0.0008 | 0.0011 | 169.33 | 0.75 | 0.45 |

**Supplementary Table 4.** Estimates of fixed effects, with hippocampal volume as outcome variable for the BETULA cohort.

|  | Estimate | Std. Error | df | t value | Pr(>\|t\|) |
| --- | --- | --- | --- | --- | --- |
| MAIN EFFECTS | | | | | |
| (Intercept) | 0.49 | 0.033 | 196.80 | 14.32 | <2e-16 |
| Education | -0.0014 | 0.0017 | 182.90 | -0.77 | 0.44 |
| Sex | 0.073 | 0.0103 | 182.10 | 7.11 | 2.5e-11 |
| Depression | -0.0072 | 0.0036 | 182.60 | -2.02 | 0.044 |
| Age_from60 | -0.0028 | 0.001 | 192.50 | -2.65 | 0.009 |
| Age_from60^2^ | -0.00008 | 0.00004 | 192.30 | -1.94 | 0.053 |
| Loneliness_medium | -0.0031 | 0.0051 | 184.80 | -0.63 | 0.53 |
| Loneliness_high | -0.021 | 0.014 | 183.20 | -1.52 | 0.13 |
| INTERACTION WITH AGE | | | | | |
| Loneliness_medium | 0.00048 | 0.00043 | 185.90 | 1.09 | 0.28 |
| Loneliness_high | 0.0019 | 0.0012 | 185.00 | 1.46 | 0.15 |

**Supplementary Table 5.** Estimates of fixed effects, with hippocampal volume as outcome variable for the BASE-II cohort.

|  | Estimate | Std. Error | df | t value | Pr(>\|t\|) |
| --- | --- | --- | --- | --- | --- |
| MAIN EFFECTS | | | | | |
| (Intercept) | 8184.46 | 223.73 | 176.19 | 36.58 | <2e-16 |
| Sex | -153.61 | 112.26 | 67.03 | -1.37 | 0.18 |
| Age_from10 | 262.77 | 93.86 | 211.50 | 2.80 | 0.006 |
| Age_from10^2^ | -20.09 | 10.52 | 212.38 | -1.09 | 0.06 |
| Negative Life Events | -38.19 | 18.07 | 174.46 | -2.11 | 0.04 |
| Loneliness_medium | -173.67 | 249.65 | 181.36 | -0.70 | 0.49 |
| Loneliness_high | 187.05 | 348.95 | 174.62 | 0.54 | 0.59 |
| INTERACTION WITH AGE | | | | | |
| Loneliness_medium | 59.37 | 54.92 | 174.96 | 1.08 | 0.28 |
| Loneliness_high | -31.50 | 73.65 | 202.36 | -0.43 | 0.67 |

**Supplementary Table 6.** Estimates of fixed effects, with hippocampal volume as outcome variable for the HUBU cohort.
